# The impact of alcohol control policy on pneumonia mortality in Lithuania: an interrupted time-series analysis

**DOI:** 10.1017/S0950268822000711

**Published:** 2022-04-20

**Authors:** Anush Zafar, Jürgen Rehm, Xinyang Feng, Huan Jiang, Kawon Victoria Kim, Jakob Manthey, Ričardas Radišauskas, Mindaugas Štelemėkas, Janina Petkevičienė, Alexander Tran, Shannon Lange

**Affiliations:** 1 Institute for Mental Health Policy Research, Centre for Addiction and Mental Health, Toronto ON, Canada; 2 School of Public Health Sciences, University of Waterloo, Waterloo ON, Canada; 3 Dalla Lana School of Public Health, University of Toronto, Toronto ON, Canada; 4 Campbell Family Mental Health Research Institute, Centre for Addiction and Mental Health, Toronto ON, Canada; 5 Institute of Clinical Psychology and Psychotherapy, Technische Universität Dresden, Dresden, Germany; 6 Department of Psychiatry, University of Toronto, Toronto ON, Canada; 7 Institute of Medical Science, University of Toronto, Toronto ON, Canada; 8 Center for Interdisciplinary Addiction Research (ZIS), Department of Psychiatry and Psychotherapy, University Medical Center Hamburg-Eppendorf (UKE), Hamburg, Germany; 9 Department of International Health Projects, Institute for Leadership and Health Management, L.M. Sechenov First Moscow State Medical University, Moscow, Russian Federation; 10 Department of Psychiatry, Medical Faculty, University of Leipzig, Leipzig, Germany; 11 Department of Environmental and Occupational Medicine, Faculty of Public Health, Lithuanian University of Health Sciences, Kaunas, Lithuania; 12 Institute of Cardiology, Lithuanian University of Health Sciences, Kaunas, Lithuania; 13 Health Research Institute, Faculty of Public Health, Lithuanian University of Health Sciences, Kaunas, Lithuania; 14 Department of Preventive Medicine, Faculty of Public Health, Lithuanian University of Health Sciences, Kaunas, Lithuania

**Keywords:** Alcohol consumption, pneumonia, infectious diseases, alcohol control policy, Lithuania

## Abstract

Despite the growing body of evidence suggesting that alcohol consumption is associated with an increased risk of and poorer treatment outcomes from pneumonia, little is known about the association between alcohol control policy and pneumonia mortality. As such, this study aimed to assess the impact of three alcohol control policies legislated in 2008, 2017 and 2018 in Lithuania on sex-specific pneumonia mortality rates among individuals 15+ years of age. An interrupted time-series analysis using a generalised additive mixed model was performed for each policy. Of the three policies, only the 2008 policy resulted in a significant slope change (i.e. decline) in pneumonia mortality rates among males; no significant slope change was observed among females. The low *R*^2^ values for all sex-specific models suggest that other external factors are likely also influencing the sex-specific pneumonia mortality rates in Lithuania. Overall, the findings from this study suggest alcohol control policy's targeting affordability may be an effective way to reduce pneumonia mortality rates, among males in particular. However, further research is needed to fully explore their impact.

## Introduction

Pneumonia, as defined by the World Health Organization (WHO), is a form of respiratory infection that causes inflammation in the lungs, limits oxygen intake and consequently makes breathing difficult [1]. Pneumonia, together with other lower respiratory infections, was considered the fourth leading cause of death worldwide in 2019 [2] and had a significant overall disease burden, with an estimated 2.5 million deaths globally and approximately 97 million disability-adjusted life years (DALYs) [3]. It is estimated that in Europe in 2003, the cost of pneumonia was approximately ten billion euros annually, with the indirect cost due to lost productivity accounting for 3.6 billion euros [4]. Current estimates of the absolute burden of pneumonia in Europe is complicated due to ongoing cost-containment efforts that have predominantly shifted pneumonia care to outpatient settings which are frequently not reported to national databases [5]. Overall, relatively high case fatality along with systematic underestimation of the full burden of disease make pneumonia a serious clinical and public health concern.

Alcohol consumption has been recognised as one of several risk factor for pneumonia [6]. The mechanism by which alcohol consumption increases the risk of pneumonia is through weakening the immune system and increasing the host's susceptibility to infection [3]. The causal pathways leading to alcohol-attributable pneumonia via compromised immunity are numerous and primarily target the key immune cells involved in combatting respiratory conditions, known as neutrophils, lymphocytes, macrophages, and other cells inherently responsible for immune responses [3]. A meta-analysis by Simou *et al*. [7] found that the risk of community-acquired pneumonia significantly increased in people who consumed alcohol compared to those who did not (relative risk (RR) = 1.61, 95% confidence interval (CI) 1.25 to 2.08), and that for every 10–20 g higher alcohol intake per day, the risk of community-acquired pneumonia increased by 8% [7].

Alcohol consumption does not only impact the aetiology of pneumonia but can also worsen its course [3], which may in turn increase the risk of death. The 2019 Global Burden of Disease Study estimated that 3.2% of deaths and 1.8% of the DALYs due to pneumonia and other lower respiratory infections were alcohol-attributable [3]. In the European Union (EU), where the highest volume of alcohol is consumed in the world [8], pneumonia was responsible for approximately 3% of all deaths in 2016 [9]. Given the association between alcohol consumption and pneumonia, strengthening alcohol control policies to reduce alcohol intake should reduce the incidence of pneumonia and thereby pneumonia mortality.

Lithuania, an EU member state, had been one of the heaviest drinking countries globally, with an estimated 16.0 litres of pure alcohol adult per capita in 2005 and 2006 [10]. In an effort to combat the increasing levels of alcohol consumption and alcohol-attributable harm, Lithuania declared 2008 the ‘year of sobriety’ and from that point onwards introduced various alcohol control policies, including the World Health Organization (WHO) ‘best buys’ (increased taxation, reduced availability and marketing bans) [11]. Of the policies implemented in Lithuania since 2000, Rehm *et al*. [12] classified those policies resulting in decreased alcohol affordability and reduced availability (i.e. those legislated January 2008, March 2017 and January 2018) as being the most likely to have an immediate effect on alcohol consumption and subsequent alcohol-attributable harm [12]. While these policies have been previously shown to significantly reduce alcohol consumption (see [13] for recorded, [14] for total adult per capita consumption, and [15] for all-cause mortality rates), it is not clear whether they had an impact on pneumonia mortality specifically. In fact, to date, no interrupted time series analysis has assessed the effect of alcohol control policies on pneumonia mortality, or pneumonia incidence for that matter. Therefore, the purpose of the current study was to examine the impact of alcohol control policies targeting affordability and availability on pneumonia mortality among individuals 15+ years of age in Lithuania. It was hypothesised that the respective alcohol control policies led to a reduction in the pneumonia mortality rate. Given that Lithuania has consistently reported a notable gap between males and females with respect to alcohol use, with 27.9 and 9.7 litres of pure alcohol per capita in 2016, respectively [8], all analyses were sex-specific.

## Methods

### Measures

Monthly sex-specific pneumonia mortality data and population data were obtained from Statistics Lithuania by the Lithuanian University of Health Sciences [16] for individuals 15+ years of age by five-year age groups from January 2001 to December 2019 (*n* = 228 months). The sex-specific number of deaths by age group was divided by the sex-specific population for each respective age group for each month, and then multiplied by 100 000 population to calculate the pneumonia mortality rate as deaths per 100 000 population. A weighted mean was then computed for all individuals 15+ years of age, using the European standard [17] to create a monthly age-standardised pneumonia mortality rate. The following International Classification of Diseases 10^th^ revision codes were used to define pneumonia deaths: A48.1, A70, J09-J15.9, J16-J16.8 J17-18.9 J20-J21.9 (See online Appendix, Table 1).

The independent variables to be tested were those policies implemented on 1 January 2008 (Policy 1), 1 March 2017 (Policy 2) and 1 January 2018 (Policy 3) [11], and were coded as dummy variables (‘0’ for all months preceding policy implementation and ‘1’ for all months following implementation). A description of the three alcohol control policies tested is presented in Table 1; for additional details see the paper by Miščikienė *et al*. [11].

**Table 1.** Detailed description of the three alcohol control policy enactments tested in the time-series models

Policy	Description	Date
Policy 1	*Stricter drinking and driving legislation:* Increased fines for drinking and driving Car confiscation and/or driver imprisonment Legally allotted blood alcohol content (BAC) reduced from 0.4 to 0.2 per mille for young drivers *Ban on alcohol advertising:* Alcohol advertising is banned on TV and radio during the day time (between 6 am and 11 pm) *Increased taxation:* Excise tax increased by 10% for beer and 20% for other types of alcohol	1 January 2008
Policy 2	*Further increase in taxation:* Excise tax increases by a large amount: 112% for beer, 111% for wine, 93% for intermediate products and 23% for ethyl alcohol	1 March 2017
Policy 3	*Stricter alcohol availability:* Legal age to purchase or consume alcohol increases from 18 to 20 years old Alcohol vendors are required to ask for identification for buyers appearing younger than 25 years old Off-site alcohol sales (i.e. not at restaurants and bars) are restricted between the hours 10 am and 8 pm, Monday to Saturday, and 10 am to 3 pm on Sundays (exceptions: airports, ferries, train bars/shops). *Further ban on alcohol advertising:* Complete ban of alcohol advertising on TV, radio, and digital ads, with exception to printed logos in sale places, producers' webpages and memorabilia	1 January 2018

### Covariates

Since alcohol consumption and alcohol-attributable harm are both influenced by economic factors, such as pricing and affordability [18], the following three covariates were considered for inclusion in the analyses: quarterly gross domestic product *per capita* (GDP), monthly consumer price index (CPI) of alcoholic beverages compared to December of the previous year, and monthly sex-specific unemployment rates. Data on GDP, CPI and unemployment rate were obtained from Statistics Lithuania by the Lithuania University of Health Sciences [16]. Linear interpolation was performed to derive monthly values from the quarterly GDP data.

### Statistical analysis

#### Segmented regression analysis

Sex-specific segmented regression analyses were performed to analyse trends of monthly pneumonia mortality rates between January 2001 and December 2019. Segmented regression analysis is a statistical technique, which identifies inflection points in the data and fits data-driven linear regression segments between the identified inflection points. Based on the visual inspection of the graph, two models (i.e. two and three inflection points) were fitted for both sexes, and AIC, BIC and adjusted R-squared values were used to compare the fits of two models and determine whether two or three inflection points would be appropriate. The use of data-driven linear regression segments makes it ideal for detecting an apparent change in trends by inspecting the slope change. The segmented regression analyses were conducted using the ‘segmented’ package in RStudio version 1.4.1717 for Windows [19].

#### Interrupted time series analysis using generalise additive mixed models (GAMM)

An interrupted time series analysis was employed by fitting a generalised additive mixed model (GAMM) to estimate the independent effect of the three alcohol control policies on sex-specific pneumonia mortality rates (i.e. three GAMM models for the three alcohol policy enactments were fit for females and males separately), while controlling for GDP, CPI and unemployment rate. In the GAMMs, seasonality of the sex-and-pneumonia-specific mortality rates was controlled for using a smoothing spline with 12 knots, denoting a monthly pattern. Correlation and cross-correlation between the sex-specific pneumonia mortality rates and each covariate (GDP, CPI and unemployment rate) were examined to account for any significant lagged effect. Each covariate was added to the model in a step-wise manner. The AIC, BIC and adjusted *R*^2^ values were used to determine the final model. As a result, CPI was eliminated from the final model and GDP and unemployment were added. Additionally, analysis of variance (ANOVA) tests were used to select significantly better fit model and to determine the inclusion of an interaction term between policy and months variable, and a quadratic effect of the months variable. To correct for autocorrelation in time series, autoregressive (AR) and moving average (MA) terms were added to the models when deemed necessary using the ‘auto.arima’ function and confirmed by assessing the respective autocorrelation function (ACF) and partial autocorrelation function (PACF) plots. Lastly, the model residuals were tested for normality using the Shapiro-Wilk test and was assessed using the residual plots against linear predicted values. All analyses were performed using RStudio Version 1.4.1717 for Windows [19].

## Results

**Fig. 1.**
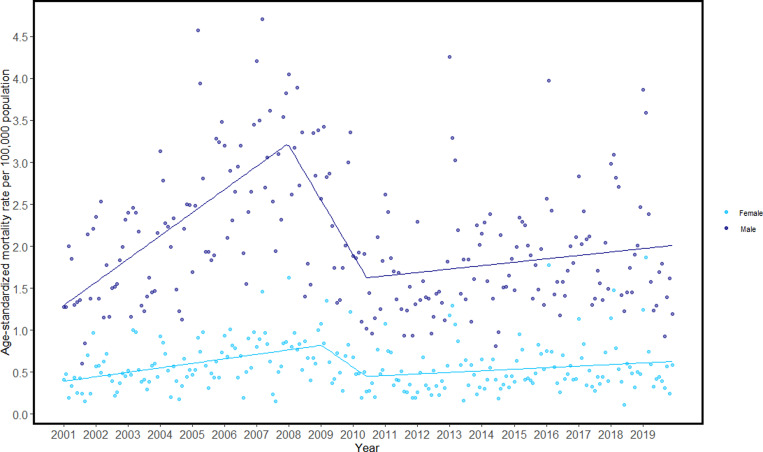
Age-standardised sex-specific pneumonia mortality rates and joinpoint trend among individuals 15+ years of age in Lithuania, 2001 to 2019.

Overall, the age-standardised pneumonia mortality rates were higher for males than for females (Fig. 1). The average annual per cent change (AAPC) for pneumonia mortality among males were 0.32% (95% CI 0.002, 0.005) and 0.1% (95% CI 0.0004, 0.002) among females. As per the segmented regression analyses, there was an overall incremental increase in pneumonia mortality rates for both sexes during the study period (Table 2). There were three periods (i.e. two change points) identified for both sexes: a significant increase for both males and females during the first period, followed by a significant decrease for males, and eventually a stagnating period for both sexes.

**Table 2.** Segmented regression analysis of age-standardised sex-specific pneumonia mortality rates (per 100 000 population) in Lithuania, 2001 to 2019

	Total study period^a^		Period 1			Period 2			Period 3		
	AAPC (%)	95% CI	Dates	Slope	95% CI	Dates	Slope	95% CI	Dates	Slope	95% CI
Males	0.0032*	0.002, 0.005	Jan. 2001 – Jan. 2008	0.023*	0.017, 0.029	Jan. 2008 Jun. 2010	−0.054*	−0.082, −0.027	Jun. 2010 – Dec. 2019	0.003	−0.0003, 0.007
Females	0.0001*	0.0004, 0.002	Jan. 2001 – Jan. 2009	0.004*	0.002, 0.006	Jan 2009 – Jun. 2010	−0.022	−0.052, 0.008	Jun. 2010 – Dec. 2019	0.002	−0.000004, 0.003

AAPC, average annual per cent change; Slope.

a Jan. 2001 – Dec. 2019.

*P < 0.05.

There was no significant effect for the three policies and pneumonia mortality rates among females. However, Policy 1 had a significant effect on pneumonia mortality among males. A slope change was observed after the implementation of Policy 1. The ‘months’ term (*β* = 0.006; CI 0.001, 0.011) and the interaction term (‘months × policy effect’: *β* = −0.009; CI −0.013, −0.005) were significant, and the resultant slope change can be determined by adding the coefficients for the two variables, which would be equal to a monthly decrease of −0.003 in pneumonia mortality after the policy implementation. Therefore, it can be inferred that with the implementation of Policy 1, a reduction in pneumonia mortality was observed among males. Additionally, there was no significant effect of Policy 2 and 3 on male pneumonia mortality rates (see Table 3). Moreover, of all the covariates, only unemployment rate was negatively associated with pneumonia mortality for both males and females at statistically significant levels. It should be noted that the adjusted-*R*^2^ values for all pneumonia mortality GAMMs were relatively low (see Table 3), suggesting that other external factors are likely influencing the sex-specific pneumonia mortality rates in Lithuania.

**Table 3.** Generalised additive mixed model regression coefficients for sex-specific pneumonia mortality rates in Lithuania, 2001–2019

	Policy 1 (1 Jan 2008)		Policy 2 (1 Mar 2017)		Policy 3 (1 Jan 2018)	
	Estimate (95% CI)	*P*	Estimate (95% CI)	*P*	Estimate (95% CI)	*P*
Males						
Adjusted *R*^2^	0.54		0.50		0.50	
Intercept	0.907 (0.576, 1.238)	<0.001*	1.010 (0.796, 1.22)	<0.001*	1.000 (0.787, 1.212)	<0.001*
Policy effect	0.659 (0.297, 1.021)	0.0004*	−0.102 (−0.260, 0.056)	0.206	−0.097 (−0.264, 0.07)	0.258
Months	0.006 (0.001, 0.011)	0.017*	−0.003 (−0.005, −0.001)	0.002*	−0.003 (−0.006, −0.002)	0.0005*
Unemployment rate	−0.032 (−0.045, −0.018)	<0.001*	−0.033 (−0.043, −0.024)	<0.001*	−0.033 (−0.042, −0.023)	<0.001*
GDP	−0.00005 (−0.0002, 0.00009)	0.511	0.0001 (0.00003, 0.0002)	0.017*	0.0001 (0.00003, 0.0002)	0.010*
Interaction term^a^	−0.009 (−0.013, −0.005)	<0.001*	n/a	n/a	n/a	n/a
Females						
Adjusted *R*^2^	0.38		0.38		0.37	
Intercept	0.208 (−0.396, 0.811)	0.500	−0.077 (−0.463, 0.308)	0.694	−0.098 (−0.481, 0.285)	0.617
Policy effect	0.491 (−0.013, 0.995)	0.058	−0.096 (−0.320, 0.128)	0.400	−0.030 (−0.265, 0.207)	0.808
Months	0.0009 (−0.007, 0.009)	0.819	−0.001 (−0.004, 0.002)	0.361	−0.002 (−0.004, 0.001)	0.223
GDP	−0.0001 (−0.0004, 0.00008)	0.231	0.00001 (−0.0001, 0.0002)	0.862	0.00002 (−0.0001, 0.0002)	0.762
Unemployment rate	−0.065 (−0.097, −0.034)	<0.001*	−0.051 (−0.073, −0.028)	<0.001*	−0.048 (−0.070, −0.027)	<0.001*
Interaction term^a^	−0.003 (−0.009, 0.003)	0.380	n/a	n/a	n/a	n/a

CPI, consumer price index; GDP, gross domestic product per capita; n/a, not applicable.

a Interaction between the months and policy effect term (dummy variable).

*P < 0.05.

## Discussion

In the current study, it was found that the age-standardised pneumonia mortality rates for both males and females have modestly increased in Lithuania from 2001 to 2019 (by 0.32% and 0.1% per year, respectively). In contrast, Marshall *et al*. [20] found that between 2001 and 2014 the pneumonia mortality rate in Lithuania increased by 6% among males and that the rate among females decreased by 8.6%. The different findings could be because of the different observation periods, data sources and the ICD-10 codes used to categorise pneumonia mortality (see [20] for details on ICD-10 codes used). Further, we found that the average annual per cent increase among males was three times higher than that among females, which indicates that the pneumonia mortality gender gap increased during the study period. Although the gender gap in pneumonia mortality in Lithuania has been consistently observed [20], the increasing gap is a novel finding.

In the current study, we hypothesised that the implementation of the alcohol control policies described above would be associated with a decrease in pneumonia mortality rates. However, Policy 1 (i.e. that implemented in 2008), which comprised a 10–20% increase in excise taxation and other measures (ban on marketing and increased penalties for drink driving) [12], was the only policy found to have had a significant negative association with pneumonia mortality, and for males only. It is worth pointing out that in Lithuania the pneumonia mortality rate is much lower among females compared to males, which may explain why no effect was found among females. It is also possible that no effect was found among females because of the stark differences in drinking behaviour between the sexes [8], as noted above. Given the well-documented gender gap in alcohol use, and association between alcohol use and pneumonia, it is likely that alcohol-related pneumonia mortality is a relatively rare outcome among females. Further, the finding that only Policy 1 had an impact was surprising. This could be due to the fact that Policy 1 included several alcohol control measures, while Policy 2 only consisted of an increase in excise taxation and, as discussed below, Policy 3 has a limited number of timepoints following it.

To the best of our knowledge, this study is the first to evaluate the association between alcohol control policies and pneumonia mortality; therefore, our ability to draw comparisons with existing studies is limited. Given this scarcity, we compare our findings with those of Štelemėkas *et al*. [15]. These investigators found an impact of all three policies on all-cause mortality. In contrast, we found that only one out of the three policies (i.e. Policy 1) was significantly associated with pneumonia mortality reductions among males. In addition to the reasons stated above, another possible explanation for the differing results could be the misclassification or underestimation of deaths due to pneumonia. A study by Mieno *et al*. [21] cross-referenced the cause of death on death certificates with the cause of death in clinical records and found that pneumonia is often misclassified.

Moreover, in our results we consistently found a significant negative association between pneumonia mortality and unemployment rate among both sexes. In line with our findings, Ruhm [22] analysed mortality fluctuations among 50 states in the US from 1972 to 1991 and found a strong inverse association between unemployment and all-cause mortality, including deaths from pneumonia, with 1% increase in the employment rate was associated with a 0.7% decrease in deaths from pneumonia and influenza. A potential explanation for such findings is that all-cause mortality, including deaths from pneumonia, tend to oscillate with the so-called business cycles, increasing with economic expansions and decreasing during recessions [22, 23]. The increase in traffic during economic upturns increases atmospheric pollution, consequently increasing the risk of deaths due to respiratory diseases, cardiovascular events and traffic collisions [23].

There are a few limitations of the current study that should be acknowledged. First, the lag structure of alcohol consumption on pneumonia mortality is not yet empirically determined. For the purpose of this analysis, a full immediate effect was assumed, based on the association between alcohol consumption and the risk of pneumonia incidence, due to the suppression of the immune system when consuming alcohol [24]. However, as pneumonia related fatalities often occur with a time lag of several weeks, especially if the patient is immunocompromised [25], the effect of alcohol control measures may be better modelled with a lag structure. Second, the adjusted-*R*^2^ values were below 55% for all models and thus, indicate that there are other factors influencing the pneumonia mortality rates in Lithuania. Further, our data comprised of relatively low pneumonia mortality rates for both sexes and even lower for females; therefore, it would be unlikely to observe very substantial changes in pneumonia mortality as any small change in deaths due to pneumonia would mean that there is a significant change in mortality due to the policy alone. Additionally, our models would have benefitted from the inclusion of other important co-variates, such as flu vaccination rates or air pollution to provide insight into the observed changes in pneumonia mortality rates. Also, due to the nature of the study design, causality cannot be inferred. Lastly, our analysis yielded the most current data, comprising data collected until 2019, which resulted in a limited number of time points following the 2018 policy. This could potentially explain the non-significant association between Policy 3 and the pneumonia mortality rate. Future research should assess the impact of Policy 3 on pneumonia mortality again when sufficient data is available.

The findings from the present study show an association between Policy 1, which involved a pricing policy that resulted in alcohol becoming less affordable, and pneumonia mortality among males. This demonstrates that alcohol control policies targeting price could be effective for reducing pneumonia mortality rates. However, since the association was not consistent (i.e. Policy 2, which also resulted in alcohol becoming less affordable, had no association with pneumonia mortality), further research in this area is necessary. Overall, our findings demonstrate the potential impact of feasible and cost-effective public health policies on general indicators, such as pneumonia mortality and, subsequently, life expectancy, which has implications for not only Lithuania but other high-income countries as well.

## Data Availability

The original data are administrative data of the Lithuanian government agencies, and need to be obtained directly from the original source (exact sources are indicated in the article).

## Supplementary material

For supplementary material accompanying this paper visit http://doi.org/10.1017/S0950268822000711. click here to view supplementary material
