# Supplementary material for: The impact of alcohol control policy on pneumonia mortality in Lithuania: an interrupted time-series analysis
Source: Epidemiol Infect. 2022 Apr 20;150:e96. doi: 10.1017/S0950268822000711 (PMC9128348; doi:10.1017/S0950268822000711)
Supplement: Supplementary file 1 [file S0950268822000711sup001.docx]

**APPENDIX**

**Table A1.** International Classification of Disease (ICD) for Pneumonia Mortality in Lithuania, 2001-2019.

| **Diseases of the Respiratory system (Pneumonia)** | **ICD-10 Codes** |
| --- | --- |
| Legionnaires' disease | A48.1 |
| Influenza due to certain identified influenza virus | J09 |
| Influenza due to other identified influenza virus | J10 |
| Influenza, virus not identified | J11 |
| Viral pneumonia, not elsewhere classified | J12 |
| Pneumonia due to Streptococcus pneumonia | J13 |
| Pneumonia due to Haemophilus influenzae | J14 |
| Bacterial pneumonia, not elsewhere classified | J15-15.9 |
| Pneumonia due to other infectious organisms, not elsewhere classified | J16-J16.8 |
| Pneumonia in diseases classified elsewhere | J17 |
| Pneumonia, organism unspecified | J18-18.9 |
| Acute bronchitis | J20 |
| Acute bronchiolitis | J21-21.9 |
| Chlamydia psittaci infections | A70 |
